# Perip7lakin is a target for autoimmunity in asthma

**DOI:** 10.1186/s12931-016-0441-5

**Published:** 2016-10-07

**Authors:** Camille Taillé, Sabine Grootenboer-Mignot, Candice Estellat, Carine Roy, Sophie Ly Ka So, Marina Pretolani, Michel Aubier, Bruno Crestani, Sylvie Chollet-Martin

**Affiliations:** 1 Assistance Publique-Hôpitaux de Paris, Hôpital Bichat, Service de Pneumologie et Centre de Compétence des Maladies Pulmonaires Rares, Paris, France; 2 Département Hospitalo-Universitaire FIRE, Université Paris Diderot, Inserm UMR 1152, LabEx Inflamex, Paris, France; 3 Assistance publique-Hôpitaux de Paris, Hôpital Bichat, Laboratoire d’immunologie « Autoimmunité et Hypersensibilités », Paris, France; 4 Assistance Publique-Hôpitaux de Paris, Hôpital Bichat, Unité de recherche clinique, département de biostatistiques, Paris, France; 5 Inserm UMR 1152, LabEx Inflamex, Paris, France; 6 INSERM UMR996, Université Paris-Sud, Université Paris-Saclay, Chatenay-Malabry, France

**Keywords:** Airway epithelium, Asthma, Autoimmunity, Desmosome, Nasal polyposis

## Abstract

The role of autoimmunity targeting epithelial antigens in asthma has been suggested, in particular in non-atopic and severe asthma. Periplakin, a desmosomal component, is involved in epithelial cohesion and intracellular signaling. We detected anti-periplakin IgG antibodies in 47/260 (18 %) patients with asthma, with no association with severity or atopy. In addition, anti-periplakin IgE antibodies were detected in 12 of 138 tested patients (8.7 %) and were more frequently observed in patients with than without nasal polyposis. This study identifies a new autoimmune epithelial target in asthma. Whether periplakin autoimmunity (both IgG and IgE auto-antibodies) is involved in asthma pathogenesis remains to be studied during the disease course of these patients.

To the Editor,

Autoimmunity targeting the bronchial epithelium has been consistently observed in patients with asthma, and various auto-antigens have been identified, including alpha enolase, [1] cytokeratine 8 or 9, [2] epidermal growth factor receptor (EGFR), activin A type 1 receptor, collagen V or α-catenin [3]. Autoantibodies (Abs) are observed in 30 % to 40 % of non-atopic patients and 0 to 10 % of atopic ones [3]. Autoimmunity may result from the exposure of self-antigens due to airway epithelial damage induced by respiratory viruses or allergens. However, the demonstration that epithelial-targeted autoimmunity contributes to asthma pathogenesis and severity is still tenuous and poorly documented: in non-atopic patients, asthma severity has been found associated with anti-collagen V Abs [3] and with the presence of IgG Abs, with a cytotoxic effect on airway epithelial cells [4].

Periplakin (PPL), a component of desmosomes, is expressed in proximal and distal airway epithelia [5]. PPL acts as a cytolinker between intermediate filament scaffolding and the desmosomal plaque and is involved in epithelial cohesion, intracellular signal transduction and antigen presentation [5]. We previously described PPL as a target for autoimmunity in idiopathic pulmonary fibrosis and that anti-PPL IgG Abs reduced alveolar cell migration in vitro [6]. Here, we hypothesized that PPL may be a target for autoimmunity in asthma and that anti-PPL IgG frequency might be different in severe compared with mild-to-moderate patients and/or in atopic compared with non-atopic patients.

We prospectively evaluated the prevalence of anti-PPL IgG Abs in the French asthma cohort COBRA (Cohorte Obstruction Bronchique et Asthme). All patients gave written informed consent for follow-up in the COBRA cohort according to the procedure validated by the local Ethics Committee (Comité de Protection des Personnes d’Ile de France 1), which approved the study.

We analysed serum obtained at enrolment in 260 patients (188 with severe and 72 mild to moderate asthma according to GINA guidelines); 40 healthy donors were used as controls. Western blot assay was used to test the presence of anti-PPL IgG Abs as previously described [6]. In 138 patients and 18 healthy donors, we also tested for the presence of anti-PPL IgE Abs. Results were considered positive when a 195-kDa band was detected with both a human placental extract and the recombinant PPL as antigenic targets. Western blot results from 6 selected patients are in Fig. 1.

**Fig. 1:**
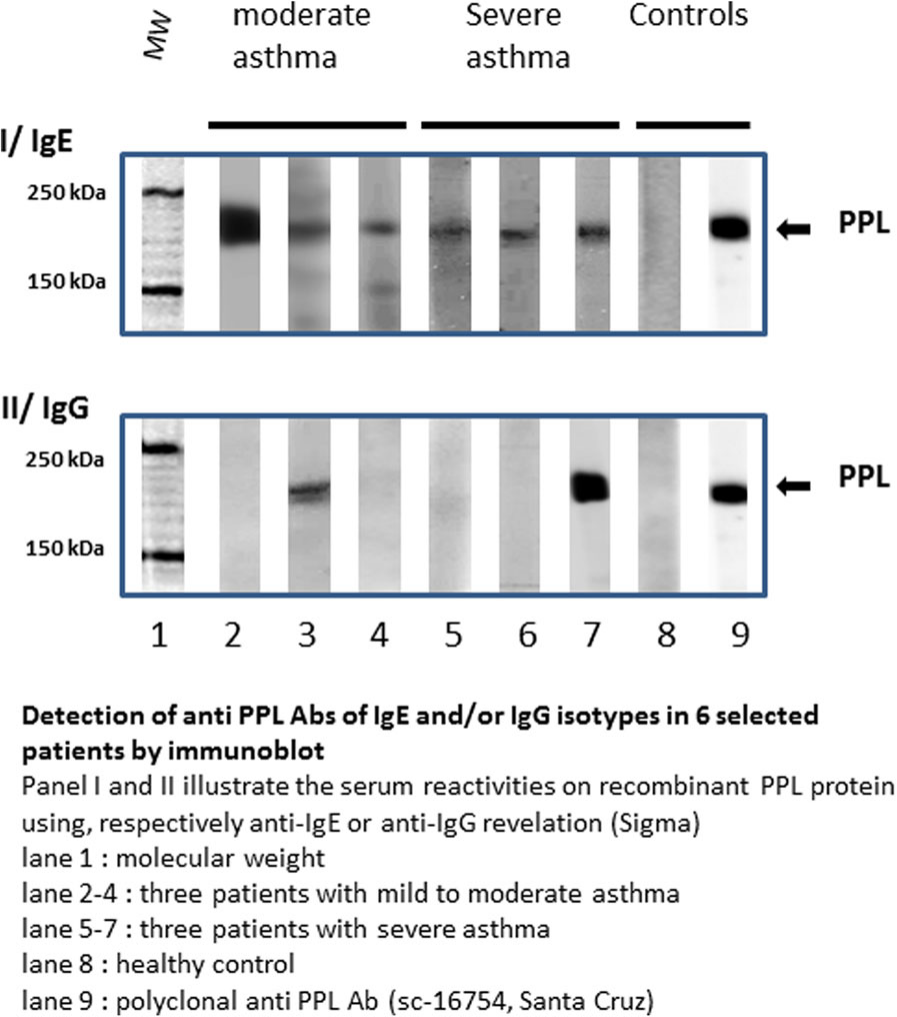
Western blot analysis of anti-periplakin (PPL) antibodies (Abs) of IgE isotype (I) and IgG isotype (II) in 6 patients. Lane 1, molecular weight; lanes 2–4, 3 patients with mild to moderate asthma; lanes 5–7, 3 patients with severe asthma; lane 8, healthy control; lane 9, polyclonal anti-PPL Ab (sc-16754, Santa Cruz Biotechnology)

Statistical analysis involved use of SAS 9.2 (SAS Inst. Inc., Cary, NC). Continuous variables were compared by Student *t* test or Wilcoxon rank-sum test and categorical variables by chi-square test or Fisher exact test, as appropriate. We tested for a differential association between anti-PPL IgG and severity or atopy with an interaction term in a multivariate logistic regression analysis explaining asthma severity. We did not adjust for multiple comparisons as it is not required in exploratory analysis [7].

Anti-PPL IgG Abs were detected in 47/260 patients (18 %) and in no control subjects. The characteristics of anti-PPL IgG–positive and –negative patients are in Table 1.

**Table 1:** Characteristics of anti-periplakin IgG and IgE –positive and –negative patients

	Total (*n =* 260)	IgG PPL + (*n =* 47)	IgG PPL– (*n =* 213)	*P* value	Total (*n =* 138)	IgE PPL + (*n =* 12)	IgE PPL– (*n =* 126)	*P* value
Male	80 (30.8 %)	20 (42.6 %)	60 (28.2 %)	0.053	44 (31.9 %)	6 (50.0 %)	38 (30.2 %)	0.19
Age (yr)	48.5 (14.2)	47.5 (13.4)	48.7 (14.4)	0.59	49.5 (15.1)	47.1 (16.3)	49.7 (15.0)	0.57
Asthma severity				0.89				0.67
Mild	35 (13.4 %)	6 (12.7 %)	29 (13.6 %)		16 (11.6 %)	2 (16.6 %)	14 (11.1 %)	
Moderate	37 (14.2 %)	8 (17.0 %)	29 (13.6 %)		16 (11.6 %)	1 (8.3 %)	15 (11.9 %)	
Severe	188 (72.3 %)	33 (70.2 %)	155(72.8 %)		106(76.8 %)	9 (75.0 %)	97 (77.0 %)	
Atopy	162 (69.8 %)	34 (77.3 %)	128(68.1 %)	0.23	95 (72.0 %)	10 (83.3 %)	85 (70.8 %)	0.5
BMI	26.9 (5.6)	26.9 (5.7)	27.0 (5.6)	0.91	27.5 (5.3)	25.4 (3.0)	27.7 (5.4)	0.14
Asthma duration (yr)	23.2 [9.1–35.9]	28.4 [14.5–37.7]	21.1 [8.0–35.7]	0.28	21.5 [8.2–36.4]	13.6 [8.0–31.7]	23.0 [8.8–36.4]	0.31
FEV1 (% predicted)	81.0 (23.4)	84.1 (20.8)	80.4 (23.9)	0.32	78.7 (24.8)	87.8 (28.2)	77.8 (24.4)	0.18
FEV1/FVC (%)	68.6 (14.4)	68.3 (15.6)	68.7 (14.2)	0.88	66.5 (15.3)	65.3 (15.0)	66.7 (15.4)	0.77
Total IgE (kUI/l)	424.0 (643.5)	446.6 (826.7)	418.6 (593.9)	0.45	425.0 (665.5)	318.2 (377.9)	436.1 (688.8)	0.82
Blood eosinophils (/mm3)	305.4 (278.8)	322.3 (185.1)	301.4 (297.1)	0.06	316.5 (251.7)	378.9 (248.0)	310.7 (252.2)	0.32
Nasal polyposis	51 (19.9 %)	13 (27.7 %)	38 (18.2 %)	0.14	27 (19.6 %)	6 (50.0 %)	21 (16.7 %)	0.01
Smoker or former smoker	100 (38.6 %)	22 (46.8 %)	78 (36.8 %)	0.26	56 (38.6 %)	4 (33.3 %)	52 (41.2 %)	0.8
Occupational asthma	32 (12.4 %)	9 (19.1 %)	23 (10.9 %)	0.12	18 (13.1 %)	1 (8.3 %)	17 (13.6 %)	1
GERD	103(39.6 %)	19 (40.4 %)	84 (39.4 %)	0.9	58 (42.0 %)	4 (33.3 %)	54 (42.9 %)	0.52
Asthma control								
well-controlled	78 (30.2 %)	17 (36.2 %)	61 (28.9 %)	0.61	38 (27.7 %)	4 (33.3 %)	34 (27.2 %)	0.92
partly controlled	76 (29.5 %)	13 (27.7 %)	63 (29.9 %)		36 (26.3 %)	3 (25.0 %)	33 (26.4 %)	
uncontrolled	104(40.3 %)	17 (36.2 %)	87 (41.2 %)		63 (46.0 %)	5 (41.7 %)	58 (46.4 %)	
JUNIPER score	10.2 (7.3)	9.1 (8.1)	10.5 (7.0)	0.29	10.4 (7.3)	6.1 (8.4)	10.8 (7.1)	0.08
At least 1 exacerbation in the previous year	147 (58.8 %)	22 (48.9 %)	125 (61.0 %)	0.13	81 (60.4 %)	8 (72.7 %)	73 (59.3 %)	0.52
No. of oral steroid bursts in the previous year	4.0 [2.0–8.0]	3.0 [2.0–4.5]	4.0 [2.0–8.0]	0.39	4.0 [2.0–8.0]	3.5 [2.0–4.0]	5.0 [2.0–8.0]	0.38
Inhaled steroids	223 (85.8 %)	42 (89.4 %)	181 (85.0 %)	0.43	123 (89.1 %)	10 (83.3 %)	113 (89.7 %)	0.62
Daily dose of inhaled steroids (μg/d)	1781.1 (1154.1)	1463.5 (1113.6)	1854.8 (1153.8)	0.01	1801.8 (1092.8)	1700.0 (1032.8)	1810.8 (1101.9)	0.86
Daily oral steroids	47 (18.1 %)	7 (14.9 %)	40 (18.9 %)	0.52	25 (18.2 %)	2 (16.7 %)	23 (18.4 %)	1
Omalizumab	15 (5.8 %)	2 (4.3 %)	13 (6.2 %)	1	8 (5.8 %)	0 (0.0 %)	8 (6.3 %)	0.36

Data are mean (SD), *n* (%) or median (interquartile range)

*PPL* periplakin, *GERD* gastroesophageal reflux disease, *FEV* forced expiratory volume in 1 s, *FCV* forced vital capacity, *BMI* body mass index Atopy was defined by a positive skin prick test and/or specific IgE level > 0.15 kU/l for at least one aeroallergen

The proportion of patients with anti-PPL IgG was similar in mild-to-moderate asthma (19.4 % [95 % CI 10.3–28.6 %]) and in severe asthma (17.6 % [95 % CI 12.6–23 %]) (*p =* 0.72). However, the association between anti-PPL IgG positivity and asthma severity differed by atopy (p_interaction_ = 0.04): anti-PPL IgG–positive non-atopic patients tended to have less severe asthma, although not significantly (odds ratio [OR] 0.26 [95 % CI 0.07–1.05], *p =* 0.059), while anti-PPL IgG-positivity was not associated with asthma severity in atopic patients (OR 1.42 [95 % CI 0.59–3.42], *p =* 0.42).

Moreover, anti-PPL IgG–positive and –negative patients only differ for the mean daily inhaled corticosteroids dose, which was lower in the positive group. Non-significant trends to a greater proportion of males, frequency of occupational asthma and nasal polyposis were observed in the anti-PPL IgG–positive group, together with a trend to a lower number of asthma exacerbations and a longer median duration of asthma.

Anti-PPL IgE Abs were detected in none of the healthy donors and in 12/138 patients (8.7 %) (Table 1); 7 were negative for anti-PPL IgG Abs. Anti-PPL IgE–positive and –negative patients did not differ in clinical characteristics, particularly atopy, except for the proportion of nasal polyposis, which was higher for anti-PPL IgE–positive than –negative patients (50 % vs 16.7 %, *p <* 0.01).

This study identifies a new epithelial target for autoimmunity in asthma. Regardless of atopic status and degree of asthma severity, about 20 % of our asthmatic patients exhibited circulating auto-Abs (IgG and/or IgE) targeting PPL. Anti-PPL IgG-positive patients received lower doses of inhaled corticosteroids, suggesting a less active disease, which is in line with a trend for a lower proportion of patients presenting an asthma exacerbation, but could also suggest a potential modulation of the anti-PPL Abs production by corticosteroids.

We consider anti-PPL Abs a marker of airway epithelium damage. Because the presence of anti-PPL IgG or IgE Abs was tested only once during the asthma time course, we cannot determine how anti-PPL autoimmunity developed during the disease course nor how it could relate to earlier periods of uncontrolled disease, beyond the 1-year period preceding inclusion. A close prospective follow-up of anti-PPL Ab–negative patients could help assess the history of anti-PPL autoimmunity, as well as the role of asthma exacerbations and inhaled/oral steroids in the development of anti-PPL Abs. A longer follow-up could also help determine whether anti-PPL–positive patients may have a different prognosis than –negative patients, for example, show a more pronounced obstructive pattern or not. The titration of Abs could also give additional information because anti-collagen V Ab levels have been found to be increased in severe asthma [3].

Our results emphasize the potential role of auto-reactive IgE (alone or associated with IgG) in asthma. Of note, anti-PPL IgE Abs were more frequent in patients with than without nasal polyposis. This is an intriguing observation because nasal polyposis is characterized by altered upper airway epithelium (loss of epithelial barrier function and loss of desmosomes) [8], as is observed in asthma. Autoreactive IgE have been described in auto immune diseases, where they can act in synergy with IgG auto-Abs to potentiate the activation of inflammatory components [9]. Most importantly, IgE auto-Abs have been also reported in atopic dermatitis [10] and in chronic urticaria [11], and are considered as potential targets for omalizumab treatment in this setting [11].

The present data do not allow for determining whether anti-PPL autoimmunity is really involved in the pathogenesis of asthma. However, since we previously showed that anti-PPL Abs inhibit epithelial repair in vitro [6], we suggest anti-PPL Abs may contribute to a vicious cycle leading to chronic airway epithelial injury, a central feature of asthma.

To conclude, in this exploratory analysis, we observed autoimmunity features (IgG and/or IgE Abs) directed against PPL, an important airway epithelial protein, in 20 % of a large cohort of patients with asthma. These results add to the controversial hypothesis about the role of autoimmunity in the pathogenesis of asthma and autoimmunity as a target for asthma treatment but also emphasize the potential role of IgE auto-Abs in asthma and nasal polyposis.

## Abbreviations

BMI: Body mass index
FCV: Forced vital capacity
FEV: Forced expiratory volume in 1 s
GERD: Gastroesophageal reflux disease
PPL: Periplakin

## References

[1] Nahm DH, Lee KH, Shin JY, Ye YM, Kang Y, Park HS (2006). Identification of alpha-enolase as an autoantigen associated with severe asthma. J Allergy Clin Immunol.

[2] Nahm DH, Lee YE, Yim EJ, Park HS, Yim H, Kang Y (2002). Identification of cytokeratin 18 as a bronchial epithelial autoantigen associated with nonallergic asthma. Am J Respir Crit Care Med.

[3] Liu M, Subramanian V, Christie C, Castro M, Mohanakumar T (2012). Immune responses to self-antigens in asthma patients: clinical and immunopathological implications. Hum Immunol.

[4] Kwon B, Lee HA, Choi GS, Ye YM, Nahm DH, Park HS (2009). Increased IgG antibody-induced cytotoxicity against airway epithelial cells in patients with nonallergic asthma. J Clin Immunol.

[5] Bouameur JE, Favre B, Borradori L (2014). Plakins, a versatile family of cytolinkers: roles in skin integrity and in human diseases. J Invest Dermatol.

[6] Taille C, Grootenboer-Mignot S, Boursier C, Michel L, Debray MP, Fagart J (2011). Identification of periplakin as a new target for autoreactivity in idiopathic pulmonary fibrosis. Am J Respir Crit Care Med.

[7] Bender R, Lange S (2001). Adjusting for multiple testing--when and how?. J Clin Epidemiol.

[8] Shahana S, Jaunmuktane Z, Asplund MS, Roomans GM (2006). Ultrastructural investigation of epithelial damage in asthmatic and non-asthmatic nasal polyps. Respir Med.

[9] Henault J, Riggs JM, Karnell JL, Liarski VM, Li J, Shirinian L (2016). Self-reactive IgE exacerbates interferon responses associated with autoimmunity. Nat Immunol.

[10] Zeller S, Rhyner C, Meyer N, Schmid-Grendelmeier P, Akdis CA, Crameri R (2009). Exploring the repertoire of IgE-binding self-antigens associated with atopic eczema. J Allergy Clin Immunol.

[11] Chang TW, Chen C, Lin CJ, Metz M, Church MK, Maurer M (2015). The potential pharmacologic mechanisms of omalizumab in patients with chronic spontaneous urticaria. J Allergy Clin Immunol.

